# Quantifying racial disparities in risk of invasive *Staphylococcus aureus* infection in Metropolitan Atlanta, Georgia, during the 2020–2021 coronavirus disease 2019 (COVID-19) pandemic

**DOI:** 10.1017/ice.2023.260

**Published:** 2024-04

**Authors:** Herveen Kaur Singh, Radhika Prakash-Asrani, Allison Pall, Susan M. Ray, Melissa Tobin-D’Angelo, Scott K. Fridkin

**Affiliations:** 1 Department of Epidemiology, Rollins School of Public Health, Emory University, Atlanta, Georgia; 2 Division of Infectious Diseases, School of Medicine, Emory University, Atlanta, Georgia; 3 Georgia Emerging Infections Program, Atlanta, Georgia; 4 Atlanta Veterans’ Affairs Health System, Atlanta, Georgia; 5 Georgia Department of Public Health, Atlanta, Georgia

## Abstract

We estimated the racial disparity in rates of invasive *S. aureus* infections based on community coronavirus disease 2019 (COVID-19) rates at the county level. Our data suggest that COVID-19 infection burden (1) affects not only hospital-onset MRSA invasive infection risk but also community-onset *S. aureus* invasive infection risk and (2) affects Black residents ∼60% more than White residents.

Incidence of invasive *Staphylococcus aureus* infections are higher among Black patients than White patients^1,2^; similar disparities were evident with *S. aureus* skin and soft-tissue infections even after adjusting place-based crowding.^3^ Racial and ethnic health disparities in coronavirus disease 2019 (COVID-19)–related outcomes are now well documented,^4^ as are associations between COVID-19–hospitalizations and some healthcare-associated infections.^5,6^ This study utilizes population-based data to estimate the impact of the COVID-19 pandemic on racial disparities in both hospital-onset and community-onset invasive *S. aureus* incidence in the 8-county Atlanta metropolitan area.

## Methods

### Data sources

The Georgia Emerging Infections Program (EIP, funded by the Centers for Disease Control and Prevention) conducts active population-based surveillance for invasive *S. aureus* infections in the 8-county metropolitan Atlanta area: Health District 3, HD3: Fulton, Dekalb, Cobb, Gwinnett, Clayton, Douglas, Newton, and Rockdale.^1^ Routine monthly reviews of reports from all area hospital microbiology laboratories and commercial laboratories are performed for patients residing in HD3. Invasive sites include normally sterile sites: blood, bone, CSF, joint, internal organs, body fluids, etc. Patient-level data (eg, demographic, clinical, susceptibility) were abstracted from patient records. COVID-19 surveillance data were obtained from GDPH through the Emory COVID-19 Response Collaborative (ECRC). These data were aggregate data, COVID-19 case counts by county, study month, and race for all confirmed COVID-19 cases in HD3. A confirmed COVID-19 case at the time of data extraction for this study was defined as an individual who had a positive polymerase chain reaction (PCR) test for COVID-19 and resided in 1 of the 8 counties. These data were downloaded from the State Electronic Notifiable Disease Surveillance Systems (SENDSS, December 4, 2022). Population denominators to calculate incidence rates were obtained from GDPH’s Online Analytical Statistical Information System (OASIS). Monthly, county-specific, race-specific populations were used in incidence calculations (Supplementary Table S1 online).

### Categorization of race

We estimated race and ethnicity-specific incidence rates of *S. aureus* and COVID-19 for mutually exclusive groups of Hispanic, non-Hispanic Black, non-Hispanic White, and other non-Hispanic cases. Race was obtained from medical records where ascertainment was performed per usual facility-specific intake procedures distinct for each healthcare network. The proportions of “unknown” race for invasive *S. aureus* cases by each covariate were small (<5%) and were not imputed for the purposes of this study. Overall, 114 invasive *S. aureus* cases (2.6%) identified as “unknown” race and were removed from the analysis.

### Statistical analyses

The *S. aureus* infection data for March 2020 through December 2021 were transformed into aggregate monthly county-specific counts. *S. aureus* data were stratified by race, susceptibility (MRSA vs MSSA), and place of infection onset, and COVID-19 data were stratified by race. Place of onset was considered “hospital onset” if infection date was >3 days after hospital admission date. Because of extreme peaks in incidence of COVID-19 infection, COVID-19 incidence was simplified for testing statistical relationships with invasive *S. aureus* incidence. This simplification was mapping the monthly COVID-19 incidence to an ordinal variable of low, low–mid, mid–high, and high incidence. These cutoff values are summarized in Supplementary Table S1 (online).

Two of the smallest counties had insufficient occurrence of invasive *S. aureus* infection and were excluded from all statistical analyses. The racial demographics of the removed counties were within the range reported from the remaining counties. The Spearman correlation test was used to evaluate the association between COVID-19 incidence quartile and invasive *S. aureus* incidence, stratified by other covariates (race, place of onset, and susceptibility). Poisson regression was used to model the association between monthly, county-level COVID-19 incidence quartile (ie, the exposure) on invasive *S. aureus* incidence (ie, the outcome). Separate models (ie, stratified Poisson regression) were used to estimate the magnitude of the association identified with each subset of the exposure (race-specific COVID-19 incidence) on outcome (eg, race-specific invasive *S. aureus* incidence) as well as overall COVID-19 incidence on other subsets of *S. aureus* infection rates (eg, hospital-onset *S. aureus*, and MRSA), accounting for county fixed effects.

### Ethics approval

This research was approved by the institutional review boards at the Atlanta VA Medical Center (data collection) and Emory University (data analysis; IRB no. MOD003-IRB00094202).

The use of COVID-19 data from the ECRC (project no. 221104) was evaluated by the DPH Institutional Review Board. This IRB determined that the use of COVID-19 data in this project was exempt from the requirement for IRB review and approval because data were aggregated counts at the county level and were considered deidentified. Efforts to post deidentified aggregate data for public access are continuing, permissions pending.

## Results

**Figure 1:**
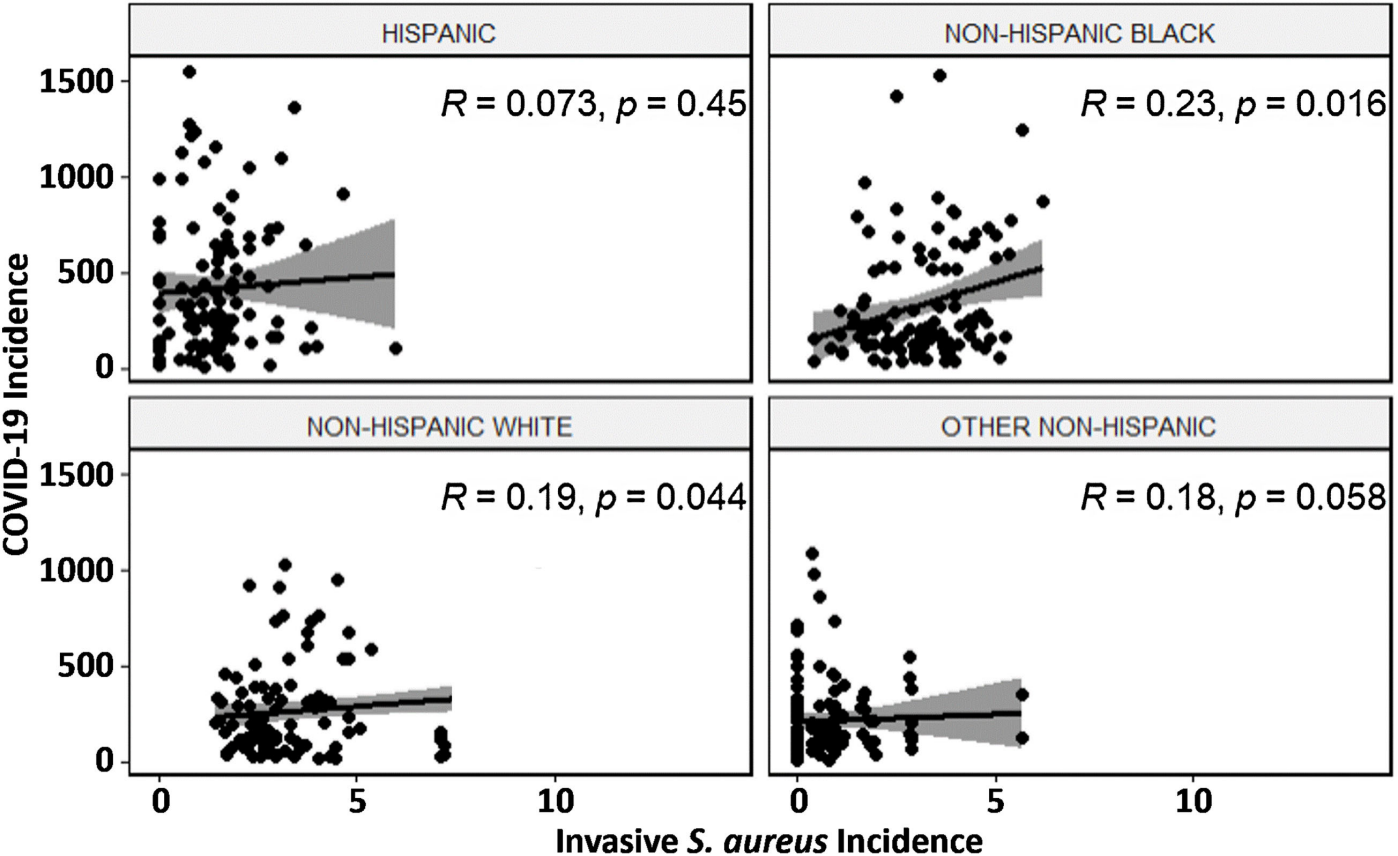
(A–D) Scatterplots and Spearman rank correlation coefficients with *P* values for county-specific monthly invasive *S. aureus* incidence and COVID-19 incidence (per 100,000 people), stratified by race for 5 counties in metropolitan Atlanta (March 2020 – December 2021). (A) Hispanic; (B) non-Hispanic Black; (C) non-Hispanic White; (D) other non-Hispanic. Note. Due to unknown values for race, 104 observations were removed.

There were 4,062 invasive *S. aureus* infections and 454,489 COVID-19 cases across all counties, with slight variation between counties (Supplementary Table S2 online). Monthly invasive *S. aureus* incidence varied between counties in any given month without any seasonal pattern; conversely, monthly COVID-19 incidence varied little between counties in any given month (Supplementary Fig. S1). Correlation between county-level monthly COVID-19 incidence and invasive *S. aureus* incidence among the 5 counties was weak but retained statistical significance among non-Hispanic Blacks (Fig. 1).

Poisson regression analysis estimated an 8% (95% confidence interval [CI], 5%–10%) increase in rate of invasive *S. aureus* incidence per quartile increase in county-specific COVID-19. For non-Hispanic Black cases, this relative increase was 9% (95% CI, 7%–11%) compared to a 5% (95% CI, −1.0% to 12%) increase among White residents. In contrast, there was no difference in the association by MRSA status, which showed an 8% increase (relative risk [RR], 1.08; 95% CI, 1.05–1.10) regardless of resistance. Hospital-onset cases increased most at 16% (95% CI, 5%–11%) per quartile increase in COVID-19 incidence (Table 1).

**Table 1.** Poisson Regression Model Stratified by Race, and Place of Onset, Fit to Predict Invasive *S. aureus* County-Level Monthly Case Counts Based on COVID-19 Quartile, Accounting for County Fixed Effects, in 5 Counties of Metropolitan Atlanta, from March 2020 to December 2021

	Model 1 (n = 110)		Model 2 (n = 440)						Model 3 (n = 220)			
	Crude		NH-White		Hispanic		NH-Black		Community		Hospital-onset	
Exposure	RR	95% CI	RR	95% CI	RR	95% CI	RR	95% CI	RR	95% CI	RR	95% CI
Quartile increase in COVID-19 incidence	1.08	1.05–1.11	1.05	0.99–1.12	1.02	0.95–1.12	1.09	1.07–1.11	1.06	1.04–1.08	1.15	1.09–1.23

Note. NH, non-Hispanic. In model 2, results of other non-Hispanic RR = 1.09 (95% CI, 0.95–1.24).

## Discussion

In this study, we quantified the impact of community COVID-19 incidence on invasive *S. aureus* infections at the county level. We estimated an ∼8% increase in infections for each step-up of COVID-19 incidence (from lowest quartile to highest quartile). When evaluating place of attribution, the effect was most impressive for hospital-onset *S. aureus* infections, such as findings by others evaluating the impact of COVID-19 hospitalization on hospital-onset MRSA bloodstream infection.^5,6^ However, our findings expand on these by quantifying this impact regardless of antibiotic susceptibility (ie, affected MRSA and MSSA similarly), and finding a significant impact on even community-onset invasive *S. aureus* infections. Secondly and notably, the magnitude of the impact was mostly due to the effect among Black patients, who experienced an increased risk of invasive *S. aureus* infection of 9% per step increase of community COVID-19 cases, whereas cases among White residents increased by only 5%.

The COVID-19 pandemic highlighted health disparities and systematic barriers regarding disease surveillance, management, prevention, and treatment.^7^ Recent analyses suggest that increases in HAIs during the COVID-19 pandemic may be attributable to 2 aspects of COVID-19 illness.^5,6,8^ At the patient level, COVID-19 patients with severe infections are exposed to medications or devices, putting patients at risk for HAIs. At the facility level, exposures occur related to breakdown in infection control, use of agency nursing staff, and changes in practice related to burnout. Also, breakdowns in infection control may pose increase risk of HAIs. Because we observed disparities after accounting for race-specific COVID-19 rates; perhaps progression of COVID-19 illness (and patient-risk for invasive *S. aureus*) differs by race leading to differential risk for invasive S. *aureus* (eg, progress to renal failure more, require central venous catheterization more). Alternatively, the facility-based exposures or differential access to care may play a more important role in the disparity, which we were unable to measure in this analysis.^8,9^

Unlike facility-based assessments evaluating the relationship between healthcare-associated *S. aureus* infections and COVID-19, our data are agnostic to the healthcare system or type of healthcare setting because they draw from regional (5 counties) surveillance efforts. Our data expand on previous findings and suggest that community COVID-19 infection burden is associated not only hospital-onset MRSA bloodstream infection risk^5,6^ but also MSSA bloodstream infection risk, as well as community-onset *S. aureus* bloodstream infection risk. Although our data include other invasive sources, ∼90% of the invasive infections have bacteremia.^1^ Moreover, this increased rate affected Black residents ∼60% more than White residents.

## Supplementary material

To view supplementary material for this article, please visit https://doi.org/10.1017/ice.2023.260
